# Postoperative complications and prognosis after radical gastrectomy for gastric cancer: a systematic review and meta-analysis of observational studies

**DOI:** 10.1186/s12957-019-1593-9

**Published:** 2019-03-18

**Authors:** Shiqi Wang, Lei Xu, Quan Wang, Jipeng Li, Bin Bai, Zhengyan Li, Xiaoyong Wu, Pengfei Yu, Xuzhao Li, Jichao Yin

**Affiliations:** 1 Xijing Hospital of Digestive Diseases, Xijing Hospital, Fourth Military Medical University, Changlexi St. 127#, Xi’An City, Shaanxi Province China; 2 Xi’an Hospital of Traditional Chinese Medicine, Fengcheng 8th St. 69#, Xi’An City, Shaanxi Province China

**Keywords:** Complications, Prognosis, Radical gastrectomy, Stomach neoplasms

## Abstract

**Background:**

Many observational studies have reported correlations between postoperative complications and prognosis after radical gastrectomy but the results are controversial. This meta-analysis was performed to investigate whether there is a correlation between postoperative complications and prognosis after radical gastrectomy.

**Methods:**

Literature searches were performed in PubMed, EMBASE, and the Cochrane Library. Studies that investigated the correlations between any postoperative complications and prognosis after radical gastrectomy were included. The pooled hazard ratio (HR) with 95% confidence interval (CI) for postoperative complications regarding overall survival (OS) or recurrence-free survival (RFS) was calculated by using RevMan 5.3.5. Subgroup analyses were performed within pathological stages I, II, and III.

**Results:**

Sixteen retrospective studies comprising 12,065 patients were included. The pooled HR (95% CI) for complications regarding OS was 1.79 (1.39, 2.30) and was 1.40 (1.06, 1.84) after excluding in-hospital mortality; the pooled HR (95% CI) for complications regarding RFS was 1.28 (1.10, 1.49). The pooled HR (95% CI) for infectious complications and leakage regarding OS was 1.86 (1.22, 2.83) and 2.02 (1.02, 4.00), respectively. The pooled HR (95% CI) for any reported postoperative complications regarding OS for stage I, II, and III diseases was 2.39 (0.77, 7.46), 4.35 (2.58, 7.35), and 2.84 (1.77, 4.56), respectively.

**Conclusions:**

Postoperative complications correlate with poor prognosis after radical gastrectomy. Such correlations are found in stage II and III gastric cancer patients but remain to be determined in stage I gastric cancer patients.

**Electronic supplementary material:**

The online version of this article (10.1186/s12957-019-1593-9) contains supplementary material, which is available to authorized users.

## Background

The incidence of postoperative complications after radical gastrectomy remains high [1–4], and the estimated incidence is 12.8 to 14% [5–7]. In addition to undermining the short-term survival, postoperative complications may also be correlated with long term prognosis. Currently, increasing numbers of observational studies have investigated the correlation between postoperative complications and long-term prognosis after radical gastrectomy. Although some reports have negative findings [8–12], other studies have demonstrated that overall postoperative complications, infectious complications, and gastrointestinal leakages are all correlated with poor overall survival (OS) and/or recurrence-free survival (RFS) [13–23]. Additionally, the correlations between postoperative complications and long-term prognosis in different stages are controversial and are based on subgroup analyses with small sample sizes [13, 18–20].

Given the prevalence of postoperative complications after radical gastrectomy, it is important to determine whether a correlation exists between postoperative complications and poor prognosis. The existence of that correlation may not only lead to a consideration of shortening follow-up interval and enforcing adjuvant chemotherapy in patient who have developed postoperative complications, but may also underline the necessity of neoadjuvant chemotherapy and stress control management in patients with high risk of developing postoperative complications to reduce the hazard for long term prognosis [9, 11, 21]. In the meta-analysis, the correlations between postoperative complications and prognosis after radical gastrectomy were assessed.

## Methods

### Search strategy and eligibility criteria

The PubMed, EMBASE, and Cochrane Library databases were searched from inception until February 24, 2019, for studies that assessed the relationship between postoperative complications and prognosis after radical gastrectomy. The following medical subject heading (MeSH) terms and keywords were used: “Stomach Neoplasms”, “Gastrectomy”, “Postoperative Complications”, and “Prognosis”. The search was restricted to studies on humans and to those that were published in the English language. The titles and abstracts were screened by two authors independently. The inclusion criterion was as follows: any study that compared the long-term prognosis between patients with and without postoperative complications after radical gastrectomy for gastric cancer. The exclusion criteria were as follows: (1) data of other neoplasms other than gastric cancer were included in the survival analysis; (2) data of palliative surgery were included in the survival analysis; (3) studies that describe the same patient population; (4) hazard ratio (HR) cannot be estimated; (5) describing complications without precise definitions; (6) letters, comments, or conference abstracts. When multiple studies describing the same patient population were identified, the most recent publication was used unless additional data were provided in the earlier work.

### Data extraction

The following data were extracted: first author, year of publication, study design, number of subjects, adjuvant chemotherapy, tumor stage, types of complications, incidences of complications, HR of any postoperative complications, and 5-year OS and 5-year RFS for patients with and without postoperative complications, as well as whether in-hospital deaths were excluded in the survival analysis. Unreported data were requested through e-mail from corresponding authors of the included studies. If there was no response to the e-mails, the missing data were estimated from the figures in the published literatures using Engauge Digitizer 4.1 (Mark Mitchell, Baurzhan Muftakhidinov, and Tobias Winchen et al., “Engauge Digitizer Software.” Webpage: http://markummitchell.github.io/engauge-digitizer) and the HRs were estimated using the method of Tierney et al. [24].

### Study quality assessment

The methodological quality of each observational study was assessed by the Newcastle-Ottawa Scale (NOS, ranging 0–9) [25]. In brief, each study was assessed for the following aspects: selection, comparability, and outcome or exposure. The comparability was primarily assessed for pathological stage and was also assessed for aspects of adjuvant chemotherapy and in-hospital death disposition in the survival analysis.

### Statistical analysis

Statistical analysis was performed with RevMan (version 5.3.5.; Cochrane Collaboration). HRs and their 95% confidence intervals (CIs) were used to evaluate the association between postoperative complications and prognosis (OS and/or RFS). Subgroup analyses were performed to investigate the correlations between infectious complication, gastrointestinal leakage, and prognosis. Furthermore, correlations were investigated for each pathological stage when possible. Statistical heterogeneities among studies were assessed by the *I*^2^ statistic. The random effects model and the fixed effects model were used. If *I*^2^ was less than 40% (cutoff point), we used the fixed effect model, while if *I*^2^ was more than 40%, the random effects model was chosen. Sensitivity analysis, in which one study was removed at a time, was performed to evaluate the stability of the results. Descriptive techniques were used when clinical heterogeneity existed or when no data could be used in the pooling analysis. The assessment of publication bias was evaluated using the funnel plot.

We followed both the Preferred Reporting Items for Systematic Reviews and Meta-Analyses (PRISMA) Statement [26], and the guidelines for Meta-analysis of Observational Studies in Epidemiology (MOOSE) in reporting this study [27]. All analyses were based on previously published studies, thus no ethical approval and patient consent are required.

## Results

### Literature searches and description of studies

**Fig. 1:**
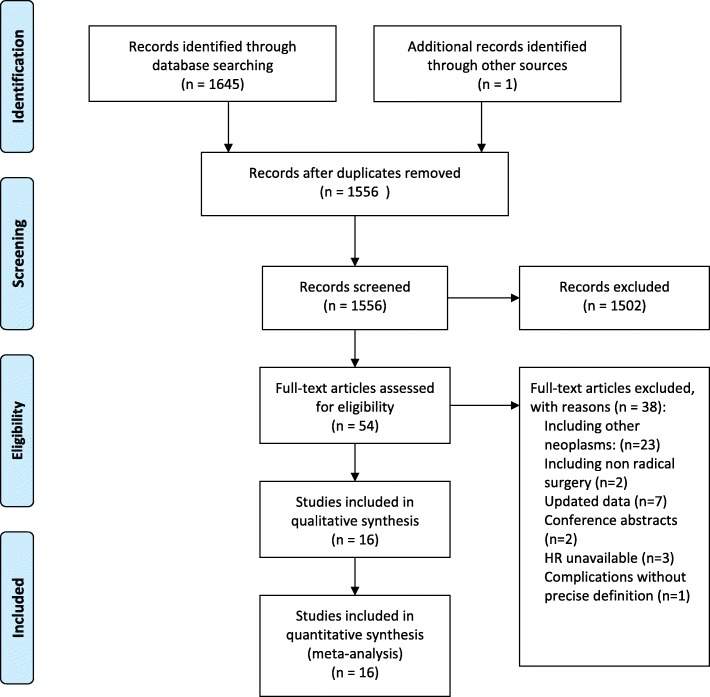
Flow chart of articles identified, included, and excluded

The flow diagram of the literature searches is shown in Fig. 1. The entire study sample size from the 16 included studies was 12,065 patients. The characteristics of the included studies are shown in Table 1. The quality of the included studies was analyzed, and the NOS scores of the included studies varied between 6 and 9 points (see Additional file 1: Table S1).

**Table 1:** Study characteristics

Author, year	NOS score	Country	Sample^a^ size	Period	Complications type	Comparable		In-hospital death	5-year RFS^b^	5-year OS^b^
						Pathological stage	Chemotherapy			
Tsujimoto et al. 2009 [13]	8	Japan	141/1191	1986–2005	Infectious	Not	Not*	Excluded	NR	57.9% vs. 78.1%
Sierzega et al. 2010 [14]	7	Poland	41/649	1999–2004	Leakage	NR	NR	Involved	NR	NR
Yoo et al. 2011 [15]	6	Korea	32/446	2000–2005	Leakage	Yes	NR	Involved	NR	9.4% vs. 68.4%
Nagasako et al. 2012 [16]	6	Japan	37/363	1997–2008	Anastomotic	NR	NR	Involved	NR	81% vs. 94.2%
Li et al. 2013 [17]	7	China	51/378	2005–2006	Overall	NR	NR	Involved	NR	21.8%vs. 39.9%
Tokunaga et al. 2013 [18]	6	Japan	81/684	2002–2006	Intra-abdominal infectious	Not	None	Involved	64.9% vs. 84.5%	66.4% vs. 86.8%
Kubota et al. 2014 [19]	7	Japan	207/981	2005–2008	Overall CD ≥ 2	Not	NR	Excluded	NR	84.1% vs. 93.1%
Hayashi et al. 2015 [20]	7	Japan	52/450	2000–2005	Infectious CD ≥ 2	Not	None	None	NR	58% vs. 83%
Kim et al. 2015 [8]	7	Korea	72/3755	2003–2012	Leakage	NR	NR	None	NR	70.8% vs. 79.3%
Saito al. 2015 [9]	6	Japan	86/219	2001–2012	Overall CD ≥ 2	NR	NR	NR	53.4% vs. 70.5%	NR
Jin et al. 2016 [21]	6	U.S.A	336/488	2000–2012	Overall	Not	Not	Excluded	23% vs. 40%	27% vs. 43%
Abdul Kader et al. 2016 [22]	7	Japan	38/227	1991–2010	Intra-abdominal	Yes	Yes	Involved	NR	24.6% vs. 69.2%
Climent et al. 2016 [10]	8	Spain	162/109	1990–2009	Overall CD ≥ 2/infectious	Yes	Yes	Excluded	46.9% vs. 54.1%	48.1% vs. 56.9%
Li et al. 2018 [23]	8	China	86/172	2008–2015	Overall CD > 2	Yes	NA	Excluded	NA	46.3% vs. 65.9%
Eto et al. 2018 [11]	9	Japan	35/66	2005–2015	Overall CD ≥ 2	Yes	Yes	None	41.7% vs. 43.9%	58.2% vs. 56.3%
Watanabe et al. 2018 [12]	7	Japan	134/296	1992–2010	Overall CD > 2	Not	Yes	Involved	46.9% vs. 45.0%	51.3% vs. 47.6%

*CD* Clavien–Dindo classification of surgical complications, *NOS* Newcastle-Ottawa Scale, *NA* not available, *NR* not reported, *OS* overall survival, *RFS* recurrence-free survival

^a^Patients number with and without concerned complications

^b^Complications group vs. control group

*More patients in the complication group received adjuvant chemotherapy

### Studies on postoperative complications and OS

**Fig. 2:**
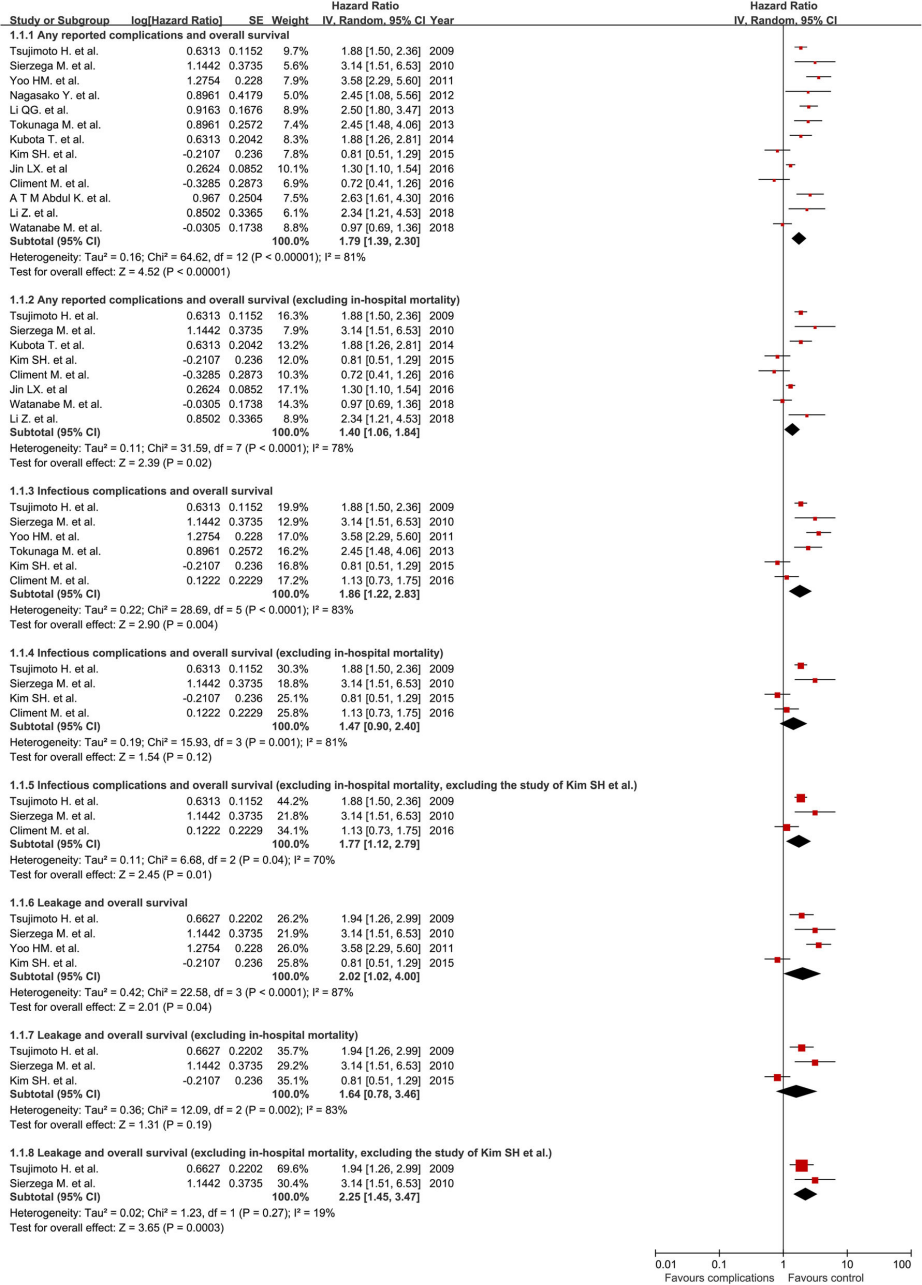
The association of postoperative complications with overall survival

**Fig. 3:**
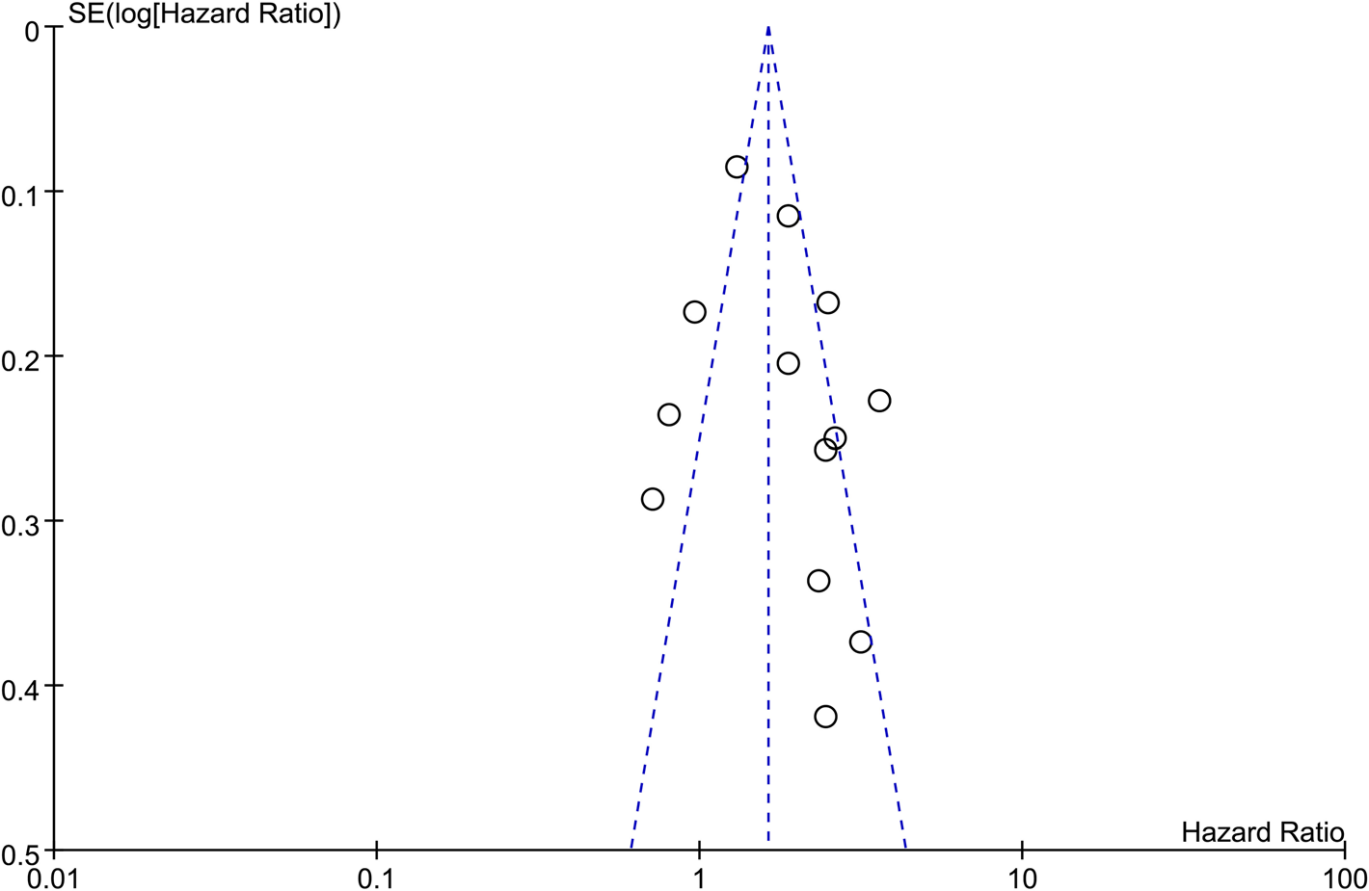
Funnel plots for visual inspection of publication bias

Thirteen studies were included in the analysis of correlation between any reported postoperative complications and OS [8, 10, 12–19, 21–23]. Of the included studies, eight excluded influences from in-hospital death in the survival analysis [8, 10, 12–14, 19, 21, 23]. The pooled HR (95% CI) of postoperative complications for OS was 1.79 (1.39, 2.30) and was 1.40 (1.06, 1.84) after excluding the in-hospital mortality (Fig. 2). The sensitivity analysis demonstrated that no individual study significantly influenced the overall effect of the HRs. Publication bias was examined by the funnel plot and there was no evidence of publication bias among these comparisons (Fig. 3).

Six studies reported the correlation between infectious complications and OS [8, 10, 13–15, 18]. Four of the studies excluded the in-hospital mortality [8, 10, 13, 14]. The pooled HR of postoperative infectious complications for OS was 1.86 (1.22, 2.83) and was 1.47 (0.90, 2.40) after excluding the in-hospital mortality (Fig. 2). Sensitivity analysis demonstrated that the study form Kim et al. caused high heterogeneity. After excluding the study, the corresponding pooled HR (95% CI) of infectious complications (in-hospital mortality excluded) changed from 1.47 (0.90, 2.40) to 1.77 (1.12, 2.79).

Four studies reported the relationship between gastrointestinal leakages and OS [8, 13–15]. Three studies excluded the in-hospital mortality [8, 13, 14]. The pooled HR of gastrointestinal leakages for OS was 2.02 (1.02, 4.00) and was 1.64 (0.78, 3.46) after excluding the in-hospital mortality (Fig. 2). Sensitivity analysis demonstrated that the study form Kim et al. caused high heterogeneity. After excluding the study, the corresponding pooled HR (95% CI) of leakage (in-hospital mortality excluded) changed from 1.64 (0.78, 3.46) to 2.25 (1.45, 3.47).

### Studies on postoperative complications and RFS

**Fig. 4:**
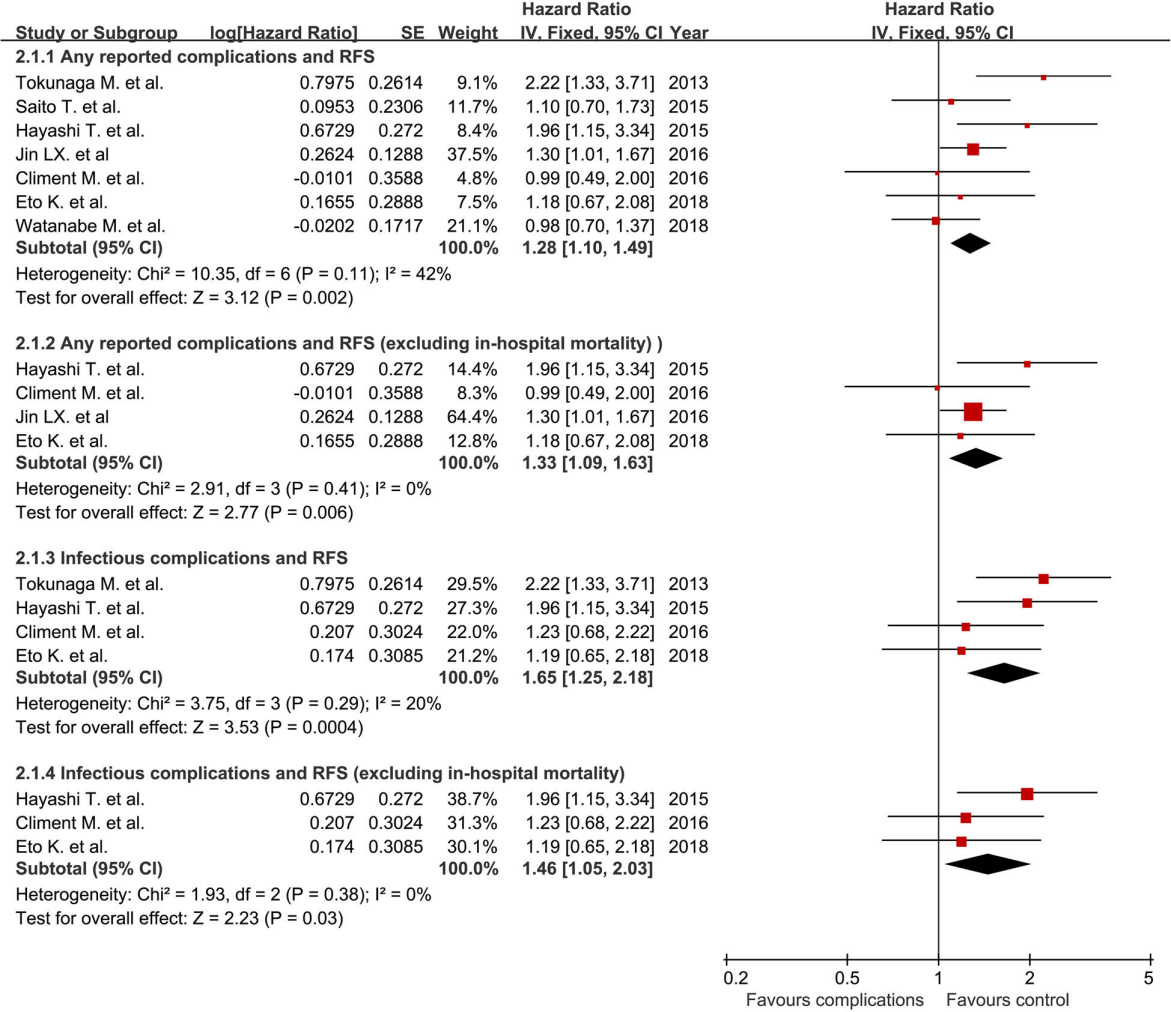
The association of postoperative complications with recurrence-free survival

Seven studies were included in the analysis of correlation between any reported postoperative complications and RFS [9–12, 18, 20, 21]. Four studies excluded the in-hospital mortality [10, 11, 20, 21]. The pooled HR for RFS is 1.28 (1.10, 1.49) and was 1.33 (1.09, 1.63) after excluding the in-hospital death (Fig. 4).

Four studies investigated the correlation between infectious complications and RFS [10, 11, 18, 20], and three of them excluded the in-hospital mortality in the analysis [10, 11, 20]. The pooled HR for the RFS in the infectious complications group was 1.65 (1.25, 2.18) and was 1.46 (1.05, 2.03) after excluding the in-hospital mortality (Fig. 4). The results of the sensitivity analysis demonstrated that no individual study significantly influenced the overall effect of HRs.

### Studies on postoperative complications and survival in separated pathological stages

**Fig. 5:**
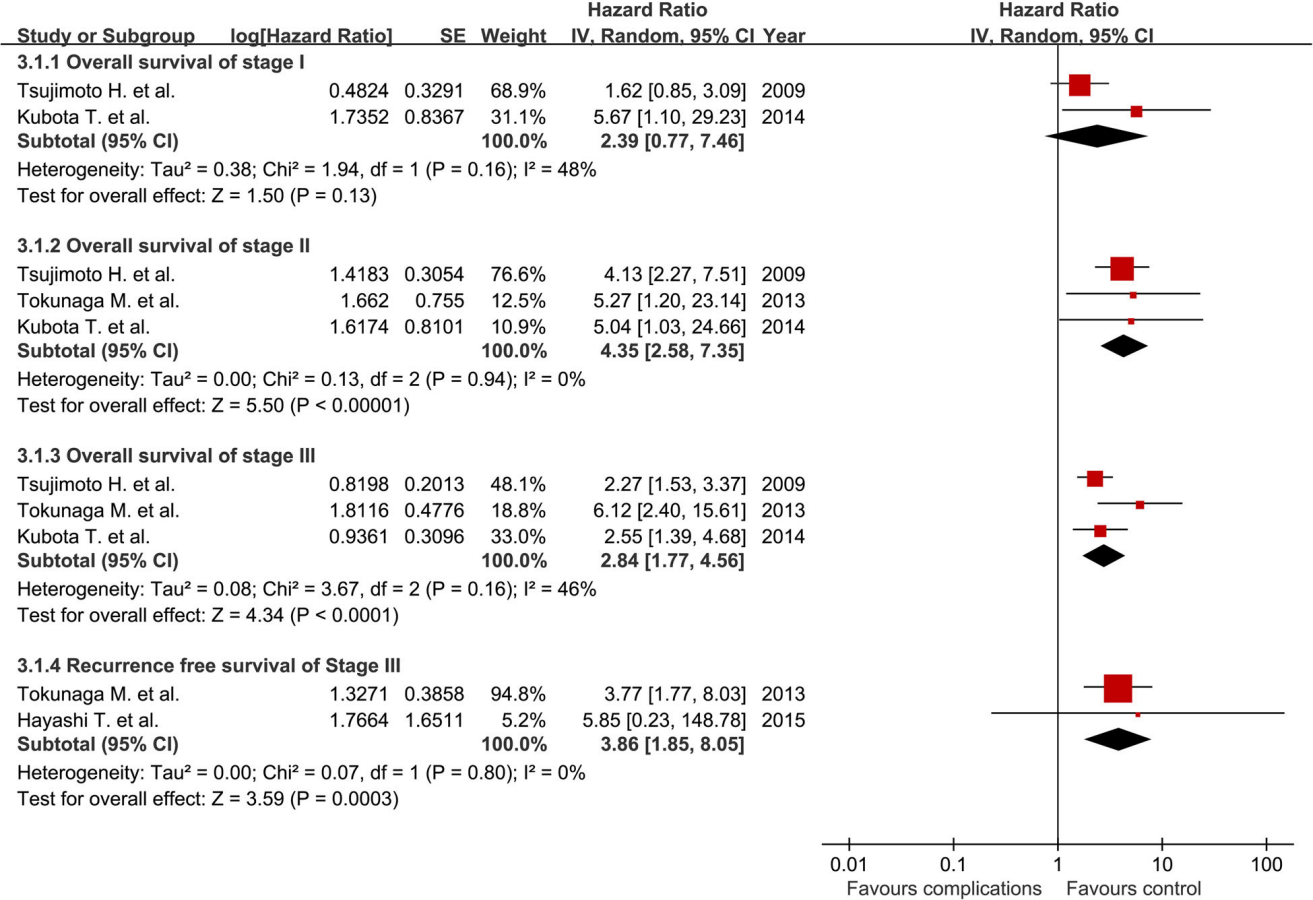
The association of postoperative complications with overall survival and recurrence-free survival within varied pathological stages

Three studies analyzed the correlations between postoperative complications and prognosis in stage I gastric cancer [13, 18, 19]. One study reported a nonsignificant correlation between postoperative complications and OS but did not present any detailed data or figures in the published report [18]. Therefore, two studies with available data were included in the analysis [13, 19]. The pooled HR (95% CI) of postoperative complications for OS in patients with stage I gastric cancer was 2.39 (0.77, 7.46) (Fig. 5).

Three studies analyzed the correlation between postoperative complications and OS in stage II gastric cancer [13, 18, 19]. The pooled HR of postoperative complications for OS in patients with stage II gastric cancer was 4.35 (2.58, 7.35) (Fig. 5).

Three studies analyzed the correlation between postoperative complications and OS in patients with stage III gastric cancer [13, 18, 19], and two studies reported the RFS [18, 20]. The pooled HR of postoperative complications for OS in patients with stage III gastric cancer from was 2.84 (1.77, 4.56), and the pooled HR (95% CI) for RFS was 3.86 (1.85, 8.05) (Fig. 5).

## Discussion

The present study undertook a comprehensive review and meta-analysis of the literatures to assess the relationship between postoperative complications and patient prognosis. The results demonstrated that, although the correlation was not found by several studies, the pooled results showed that postoperative complications correlated with poor prognosis.

Several reasons may contribute to the divergences. First, the negative findings in some studies may be ascribed to the interfered application of adjuvant chemotherapy. Jin et al. demonstrated lower proportion of adjuvant chemotherapy in the complication group (47% vs. 61%), and the combination of postoperative complications and receiving no adjuvant therapy significantly increased the hazard of death and recurrence. Furthermore, decreased OS and RFS were not observed in patients who experienced complications but received adjuvant therapy [21]. Another study demonstrated that the adjuvant chemotherapy was postponed in patients with intra-abdominal complications (55.3 ± 34.7 vs. 26.6 ± 11.9 days) [22], and the postponed chemotherapy is correlated with poorer survival in patients with gastric cancer [28]. Second, the application of prophylactic neoadjuvant chemotherapy may abolish the poor prognosis induced by postoperative complications. In a cohort with 101 patients who underwent curative gastrectomy after receiving neoadjuvant chemotherapy, Eto et al. demonstrated a comparable RFS between patients with and without postoperative complications, and the 5-year RFS was 41.7% and 43.9%, respectively [11]. Third, the varied perioperative stress level may be an additional reason for the negative finding. Saito et al. demonstrated that the postoperative inflammation degree (reflected by the CRP level), rather than the postoperative complication itself, is related to the recurrence and poor prognosis [9]. Besides, Watanabe et al. also demonstrated comparable prognosis between patients with and without postoperative complications [12]. Their patients underwent total gastrectomy with splenectomy for the treatment of proximal advanced gastric cancer. The extensive resection might lead to an excessive surgical stress in both groups and that may lead to a deteriorated prognosis in patients without postoperative complications [29].

Accordingly, the results of the present study may highlight the importance of both adjuvant and neoadjuvant chemotherapy in patients with postoperative complications or with a high risk of developing postoperative complications. The results of the present study may have also highlighted the stress control management during the perioperative period. However, whether a decreased stress level will result to an improved prognosis remains to be determined. Additionally, any other methods that decrease the postoperative complications may also indirectly improve the prognosis. The intraoperative manipulation, such as the choice of reconstruction or the less invasive approach, may play a role in decreasing the postoperative complications and thereby improve the prognosis indirectly. For instance, recent studies demonstrated that BI reconstruction method significantly reduced the postoperative complications after laparoscopic distal gastrectomy [30, 31]. Therefore, patients may benefit more from that approach with low risk of postoperative complications.

In the analysis of the relationship between infectious complications or gastrointestinal leakages and OS, the study from Kim et al. demonstrated high heterogeneity. Kim et al. found that gastrointestinal leakage was not associated with decreased survival. There are some possible reasons for the negative results [8]. First, the effect of leakage may be diluted by the effect of other complications occurred in the control group. That is to say, other complications other than gastrointestinal leakage may also contribute to the poor prognosis and that may cause an underestimated effect of leakage on prognosis. Second, the sample size may not be adequate to detect the significant correlation because their Kaplan-Meier curve demonstrated a trend of poor OS in the leakage group (*p* = 0.076) [8].

The present study had some limitations. First, five of the included studies did not exclude in-hospital death in the survival analysis [15–18, 22]. It is well acknowledged that in-hospital mortality would be higher in patients with postoperative complications and would decrease the OS accordingly. Therefore, a subgroup analysis with the eight reports that excluded in-hospital death or have no in-hospital death was performed and a similar result was found (HR 1.40, 95% CI 1.06–1.84). Second, more preoperative comorbidity, a higher ASA or ECOG score, and older age were frequently observed in the complication group, as shown in Additional file 1: Table S1, and such characteristics are correlated with a shorter life expectancy after surgery. As a result, we analyzed the data from seven studies that reported RFS [9–12, 18, 20, 21], and the HR demonstrated a positive correlation between postoperative complications and reduced RFS (HR 1. 28, 95% CI 1.10–1.49). The correlation between postoperative complications and poor RFS still exist after the in-hospital mortality were excluded (HR 1.33, 95% CI 1.09–1.63). Third, patients in the complication group frequently had more advanced disease. Eleven of the studies demonstrated the proportion of each stage, and six of the studies reported comparable stages between the two groups [10–12, 15, 22, 23]. Such a bias may cause an overestimated correlation of postoperative complications with long-term prognosis. To avoid the influence of unbalanced tumor stages, the correlations between complications and prognosis were analyzed in separate stages based on the data from four studies [13, 18–20]. In addition to the correlation between postoperative complications and decreased OS and RFS in stage II and III patients, attention should be paid to stage I patients with postoperative complications because of the undetermined result (Fig. 5). If such a correlation did exist, the application of adjuvant chemotherapy might be expanded to stage I patients who have developed postoperative complications. However, a limited number of studies were included in the subgroup analysis of separated pathological stages and the confounders cannot be avoided in the subgroup analysis. More solid evidence from studies with larger sample sizes is warranted, and RFS analysis should also be considered in further studies.

## Conclusions

In summary, there is good evidence to support the correlations between postoperative complications and poor prognosis after radical gastrectomy. The influence of postoperative complications on prognosis is also demonstrated in patients with stage II and III gastric cancer but remains to be determined in patients with stage I gastric cancer. To reduce the negative impact of postoperative complications on the long term prognosis, neoadjuvant chemotherapy may be considered in patients with high risk of developing postoperative complications and adjuvant chemotherapy should be enforced in patients who have developed postoperative complications. Additionally, perioperative stress control management might be beneficial for improving the long term prognosis after radical gastrectomy.

## Additional file

Additional file 1: **Table S1.** NOS of Cohort studies. NOS of Case-control studies. (DOCX 27 kb)

## Abbreviations

HR: Hazard ratio
OS: Overall survival
RFS: Recurrence free survival
