# Supplementary material for: Postoperative complications and prognosis after radical gastrectomy for gastric cancer: a systematic review and meta-analysis of observational studies
Source: World J Surg Oncol. 2019 Mar 18;17:52. doi: 10.1186/s12957-019-1593-9 (PMC6423865; doi:10.1186/s12957-019-1593-9)
Supplement: Supplementary file 1 — Table S1. NOS of Cohort studies. NOS of Case-control studies. (DOCX 27 kb) [file 12957_2019_1593_MOESM1_ESM.docx]

**Table S1. NOS of Cohort studies**

| Criteria | Tsujimoto, H. et al. 2009^[13]^ | Sierzega M. et al. 2010^[14]^ | Yoo, H. M. et al. 2011^[15]^ | Nagasako, Y. et al. 2012^[16]^ | Li, Q. G. et al. 2013^[17]^ | Tokunaga, M. et al. 2013^[18]^ | Kubota, T. et al. 2014^[19]^ | Jin, L. X. et al. 2016^[21]^ | Hayashi, T. et al. 2015^[20]^ | Kim, S. H. et al. 2015^[8]^ | Saito, T. et al. 2015^[9]^ | Abdul Kader, A. T. M. et al. 2016^[22]^ | Climent M. et al. 2016^[10]^ | Eto K. et al. 2018^[11]^ |
| --- | --- | --- | --- | --- | --- | --- | --- | --- | --- | --- | --- | --- | --- | --- |
| **Selection** |  |  |  |  |  |  |  |  |  |  |  |  |  |  |
| **1. Representativeness of the exposed cohort** |  |  |  |  |  |  |  |  |  |  |  |  |  |  |
| a) truly representative of the average age and sex in the community* | 1 | 1 |  |  | 1 |  |  | 1 | 1 | 1 |  |  | 1 |  |
| b) somewhat representative of the average age and sex in the community* |  |  | 1 | 1 |  | 1 | 1 |  |  |  | 1 | 1 |  | 1 |
| c) selected group of users e.g. nurses, volunteers |  |  |  |  |  |  |  |  |  |  |  |  |  |  |
| d) no description of the derivation of the cohort |  |  |  |  |  |  |  |  |  |  |  |  |  |  |
| **2. Selection of the non exposed cohort** |  |  |  |  |  |  |  |  |  |  |  |  |  |  |
| a) drawn from the same community as the exposed cohort* | 1 | 1 | 1 | 1 | 1 | 1 | 1 | 1 | 1 | 1 | 1 | 1 | 1 | 1 |
| b) drawn from a different source |  |  |  |  |  |  |  |  |  |  |  |  |  |  |
| c) no description of the derivation of the non exposed cohort |  |  |  |  |  |  |  |  |  |  |  |  |  |  |
| **3. Ascertainment of exposure** |  |  |  |  |  |  |  |  |  |  |  |  |  |  |
| a) secure record (eg surgical records) * | 1 | 1 | 1 | 1 | 1 | 1 | 1 | 1 | 1 | 1 | 1 | 1 | 1 | 1 |
| b) structured interview* |  |  |  |  |  |  |  |  |  |  |  |  |  |  |
| c) written self report |  |  |  |  |  |  |  |  |  |  |  |  |  |  |
| d) no description |  |  |  |  |  |  |  |  |  |  |  |  |  |  |
| **4. Demonstration that outcome of interest was not present at start of study** |  |  |  |  |  |  |  |  |  |  |  |  |  |  |
| a) yes* | 1 | 1 |  |  |  | NR | NR | 1 | 1 | 1 | NR |  | 1 | 1 |
| b) no |  |  | x | x | x | NR | NR |  |  |  | NR | x |  |  |
| **Comparability** |  |  |  |  |  |  |  |  |  |  |  |  |  |  |
| **1. Comparability of cohorts on the basis of the design or analysis** |  |  |  |  |  |  |  |  |  |  |  |  |  |  |
| a) study controls for pathological stage (select the most important factor) * | 1 | NR | x | x | 1 | x | x | x | x | x | x | 1 | 1 | 1 |
| b) study controls for any additional factor adjuvant chemotherapy, starting adjuvant chemotherapy, in hospital death* | x | NR | NR | x | x | x | x | x | x | x | x | x | x | 1 |
| **Outcome** |  |  |  |  |  |  |  |  |  |  |  |  |  |  |
| **1. Assessment of outcome** |  |  |  |  |  |  |  |  |  |  |  |  |  |  |
| a) independent blind assessment* |  |  |  |  |  |  |  |  |  |  |  |  |  |  |
| b) record linkage* | 1 | 1 | 1 | 1 | 1 | 1 | 1 |  | 1 | 1 | 1 | 1 | 1 | 1 |
| c) self report |  |  |  |  |  |  |  |  |  |  |  |  |  |  |
| d) no description |  |  |  |  |  |  |  | 1 |  |  |  |  |  |  |
| **2. Was follow-up long enough for outcomes to occur** |  |  |  |  |  |  |  |  |  |  |  |  |  |  |
| a) yes (≥ 12 months) * | 1 | 1 | 1 | 1 | 1 | 1 | 1 | 1 | 1 | NR | 1 | 1 | 1 | 1 |
| b) no |  |  |  |  |  |  |  |  |  |  |  |  |  |  |
| **3. Adequacy of follow up of cohorts** |  |  |  |  |  |  |  |  |  |  |  |  |  |  |
| a) complete follow up - all subjects accounted for* |  | 1 | 1 | 1 | 1 | 1 | 1 | 1 | 1 | 1 | 1 | 1 | 1 | 1 |
| b) subjects lost to follow up unlikely to introduce bias - small number lost* | 1 |  |  |  |  |  |  |  |  |  |  |  |  |  |
| c) follow up rate < 90% and no description of those lost |  |  |  |  |  |  |  |  |  |  |  |  |  |  |
| d) no statement |  |  |  |  |  |  |  |  |  |  |  |  |  |  |
| **Total score** | 8 | 7 | 6 | 6 | 7 | 6 | 6 | 7 | 7 | 6 | 6 | 7 | 8 | 9 |

If the criterion followed by an asterisk is met, the study is awarded one point. A maximum of one point can be given for each numbered item within the Selection and Outcome categories, and a maximum of two points can be given for Comparability.

**Table S1. NOS of Case-control studies**

| Criteria | Li Z. et al. 2018^[23]^ | Watanabe M. et al. 2018^[12]^ |
| --- | --- | --- |
| **Selection** |  |  |
| 1. Is the case definition adequate? |  |  |
| a) yes, with independent validation* | 1 | 1 |
| b) yes, eg record linkage or based on self reports |  |  |
| c) no description |  |  |
| 2. Representativeness of the cases |  |  |
| a) consecutive or obviously representative series of cases* | 1 | 1 |
| b) potential for selection biases or not stated |  |  |
| 3. Selection of Controls |  |  |
| a) community controls* |  |  |
| b) hospital controls | x | x |
| c) no description |  |  |
| 4. Definition of Controls |  |  |
| a) no history of disease (endpoint)* | 1 | 1 |
| b) no description of source |  |  |
| **Comparability** |  |  |
| 1. Comparability of cases and controls on the basis of the design or analysis |  |  |
| a) study controls for pathological stage* | 1 | 0 |
| b) study controls for any additional factor * (adjuvant chemotherapy, starting adjuvant chemotherapy, in hospital death) | 1 | 1 |
| **Exposure** |  |  |
| 1. Ascertainment of exposure |  |  |
| a) secure record (eg surgical records) * | 1 | 1 |
| b) structured interview where blind to case/control status* |  |  |
| c) interview not blinded to case/control status |  |  |
| d) written self report or medical record only |  |  |
| e) no description |  |  |
| 2. Same method of ascertainment for cases and controls |  |  |
| a) yes* | 1 | 1 |
| b) no |  |  |
| 3. Non-Response rate |  |  |
| a ) same rate for both groups* | 1 | 1 |
| b) non respondents described |  |  |
| c) rate different and no designation |  |  |
| **Total score** | 8 | 7 |
